# Sex and Death in the Male Pea Aphid, *Acyrthosiphon pisum*: The Life-History Effects of a Wing Dimorphism

**DOI:** 10.1673/031.007.4501

**Published:** 2007-08-21

**Authors:** Coralynn Sack, David L. Stern

**Affiliations:** Department of Ecology & Evolutionary Biology, Princeton University, Princeton, NJ, 08544, USA; ^a^ 235 Beaver Dam Rd., Brookhaven, NY 11719, USA

**Keywords:** wing polymorphism, life-history tradeoff, reproductive tactics

## Abstract

Insect dispersal dimorphisms, in which both flight-capable and flightless individuals occur in the same species, are thought to reflect a balance between the benefits and costs of dispersal. Fitness costs and benefits associated with wing dimorphism were investigated in the male pea aphid, *Acyrthosiphon pisum* (Harris) (Hemiptera: Aphididae). In one-on-one mating competitions in small arenas between winged and wingless males, the winged aphids obtained most of the matings with virgin females. In contrast, during competition experiments in larger cages with multiple individuals of each morph, the winged males no longer had a clear mating advantage over wingless males. In the absence of competition, wingless males had marginally higher lifetime reproductive success than winged males, probably because mating winged males tended to die faster than wingless males. In the absence of females, winged males survived longer than wingless males and this difference disappeared under starvation conditions. Mating males of both morphs died significantly faster than males without access to females. There does not appear to be a direct tradeoff of dispersal ability with life history characteristics in pea aphid males, suggesting that the advantages of producing winged males may result from outbreeding.

## Introduction

The widespread existence of wing polymorphisms in different insect species implies that there are tradeoffs between dispersal ability and components of fitness (Harrison 1980). Fully winged individuals benefit from the ability to colonize new areas through both long distance migration and habitat hopping within a heterogeneous environment. Flight is thought to facilitate the search for food and mates, especially within patchy environments.

However, the ability to fly may carry associated fitness costs. This hypothesis has been supported by numerous studies that compared fitness components between female wing morphs. Specifically, the maintenance of flight apparatus is energetically expensive, requiring the allocation of resources that could be used for reproduction. Flightless females often have enlarged ovaries, earlier ovarian development and reduced stores of flight fuels (Zera and Denno 1997). It is also likely that dispersers have higher mortality in natural conditions (Ward et al. 1998). Individuals that migrate, especially those that travel long distances, risk not finding a suitable habitat and may face an increased probability of predation.

Compared to females, males devote few resources to gametes. It might be thought, therefore, that allocation of resources towards the development and maintenance of flight apparatus in males may not result in large reproductive costs. However, many studies have shown that winged males exhibit the same fitness trade-offs as their female winged counterparts. In planthoppers, wingless males are more aggressive than winged males in male-male interactions, develop more quickly, acquire matings more successfully, and live longer as adults (Denno and Roderick 1990; Ichikawa 1982; Langellotto et al. 2000; Novotny 1995; Socha 2004; Socha and Zemek 2004a; Socha and Zemek 2004b). Similar results were found for water striders, bruchid beetles, chinch bugs, thrips and firebugs (Crespi 1988; Fujisaki 1992; Kaitala and Dingle 1993; Utida 1972). Furthermore, females preferentially mated with wingless males in several natural populations of water striders (Fairbairn and Preziosi 1996). In fire bugs, the mating advantage of the male morphs is age dependent (Socha 2006). These mating advantages may result from reduced development of flight muscultature, rather than from winglessness *per se* (Socha and Sula 2006; Zera et al. 1997).

In contrast, studies of a species of planthopper and two species of crickets have found no significant differences between male wing form for various components of reproductive success (Holtmeier and Zera 1993; Mishiro et al. 1994). Roff and Fairbairn (1991) suggested that wing variation among males may be an artifact of a genetic correlation between the sexes, but it seems unlikely that such complex polymorphisms would be maintained in males if they engendered no direct fitness advantage. One potential problem is that even if reproductive costs exist in the natural environment, they may be masked in laboratory experiments. Zera and Denno (1997) hypothesize that the differential ability of male morphs to locate or attract a female may not be evident in confined laboratory settings.

Aphids display dramatic wing dimorphisms. While in most insect taxa the wing polymorphism consists of differences in wing size or the extent of thoracic musculature, in aphids one morph has full flight capacity and the alternative morph is wingless (Harrison 1980). In the parthenogenic female pea aphid, *Acyrthosiphon pisum* (Harris) (Hemiptera: Aphididae), the wing dimorphism is a polyphenism in which environmental factors determine whether wing development is initiated. In contrast, the wing dimorphism in the male *A. pisum* is a genetically determined polymorphism (Braendle et al. 2005; Caillaud et al. 2002; Smith and MacKay 1989).

The adaptive significance of aphid female polyphenism has been well documented. The winged females take longer to reach sexual maturity and they produce smaller and fewer offspring (Dixon 1985). Overall, wingless aphid females can have up to 70% greater reproductive output than winged aphid females (Müller et al. 2001). The environmental determination of wing form in parthenogenic females allows aphid colonies to exhibit high fecundity while maintaining the ability to produce the less fecund winged morphs (Müller et al. 2001). Under normal conditions, the parthenogens produce the wingless nymphs, but when habitats deteriorate, environmental cues stimulate the production of the winged morphs. Host plant quality, increasing aphid density, and interactions with predators can all induce wing production in embryos (Müller et al. 2001).

There have been no reports on the fitness tradeoffs, if any, associated with the male wing dimorphism in pea aphids, although the maintenance of this genetic polymorphism in nature suggests that there are trade-offs between life history parameters (Smith and MacKay 1989). We examined life history characteristics of the male winged and wingless pea aphid. Individual fecundity in the absence of competition with other males, survivorship, individual mating ability in one-on-one competitions, and mating success were measured in small cages with several individuals of each morph competing over a longer period of time.

## Methods and Materials

### Rearing conditions

Colonies of *A. pisum* were reared on alfalfa, *Medicago sativa*. Sexual morphs were obtained by exposing asexual females to “autumnal” conditions (13:11 L:D, 15° C). Third and fourth instar aphids were isolated in Petri dishes (60 × 15mm; Falcon) containing one leaf of moon trefoil, *Medicago arborea*, (replaced when necessary) inserted into 3ml of 2% agar containing g/L plant fertilizer (Miracle Gro, www.miraclegro.com). These are henceforth referred to as *“Medicago* plates”. Newly molted adults were allowed to mature for twenty-four to forty-eight hours, at which point these virgins were used for experiments.

### Lifetime fecundity experiments

Lifetime male fecundity was estimated for both winged and wingless males by providing individual males with a constant supply of females. 24 to 48 hour old virgin adult males were isolated with four virgin females in a *Medicago* plate. Every 48 hours, these four females were replaced by four new virgin females. This process was repeated until the male died. The male genitalia were dusted with a pink powder, which allowed us to determine whether a female had been mated by examining her external genitalia for the presence of the powder. In preliminary experiments, the powder was transferred from the male genitalia to the female during attempted mating and the powder persisted on individuals after multiple matings. Numbers of matings per male were included in the analysis only for males surviving to the end of each survey period.

Frequency of mating does not necessarily reflect reproductive fitness, especially if copulations are not always successful. Therefore the number of females that each male fertilized was determined by isolating females from this experiment until they produced eggs. Fertilized eggs turn black 3 to 5 days after they have been laid (Miura et al. 2003).

For each of the 30 winged and 15 wingless replicate trials, differences in survivorship between the winged and wingless morph were assessed using the Mann-Whitney *U* test (Pyke and Thompson 1986). Age-specific fecundity was calculated both as the average number of females mated and the average number of females fertilized by a male at age x. The Mann-Whitney *U* Test was used to determine if there was a difference in the mean daily mating and fertilization rates between the two morphs (Sokal and Rohlf 1995). The cumulative reproductive rate (R_0_ for the number of females mated and number of females fertilized) was calculated according to the formula:

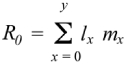

where x is the age class, l_x_ is survival rate at a specific age class, and m_x_ is the average number of females mated or fertilized at a specific age class. The Mann-Whitney *U* Test was used to determine if there was a difference in cumulative reproductive fecundity between winged and wingless males. Statistical analyses were implemented with JMP 5.1.

### Comparative survivorship of male wing forms

While the fecundity experiment provided an overview of potential tradeoffs between the morphs, to gain a more detailed understanding of the potential tradeoffs further experiments were done on male survival and mating ability in a variety of laboratory conditions. First, the longevity of the two male wing morphs was measured in the presence and absence of food. In the first experiment, with food available, males (80 winged and 47 wingless) were maintained individually in *Medicago* plates. In the second experiment, assessing survivorship without food, males (55 winged and 34 wingless) were maintained individually in Petri dishes (60 × 15mm) with a moist piece of filter paper. Survivorship curves were generated for both wing morphs in each experiment by calculating the proportion of the initial cohort surviving each day. Differences between survivorship curves were assessed using the Mann-Whitney *U* test.

**Figure 1.**
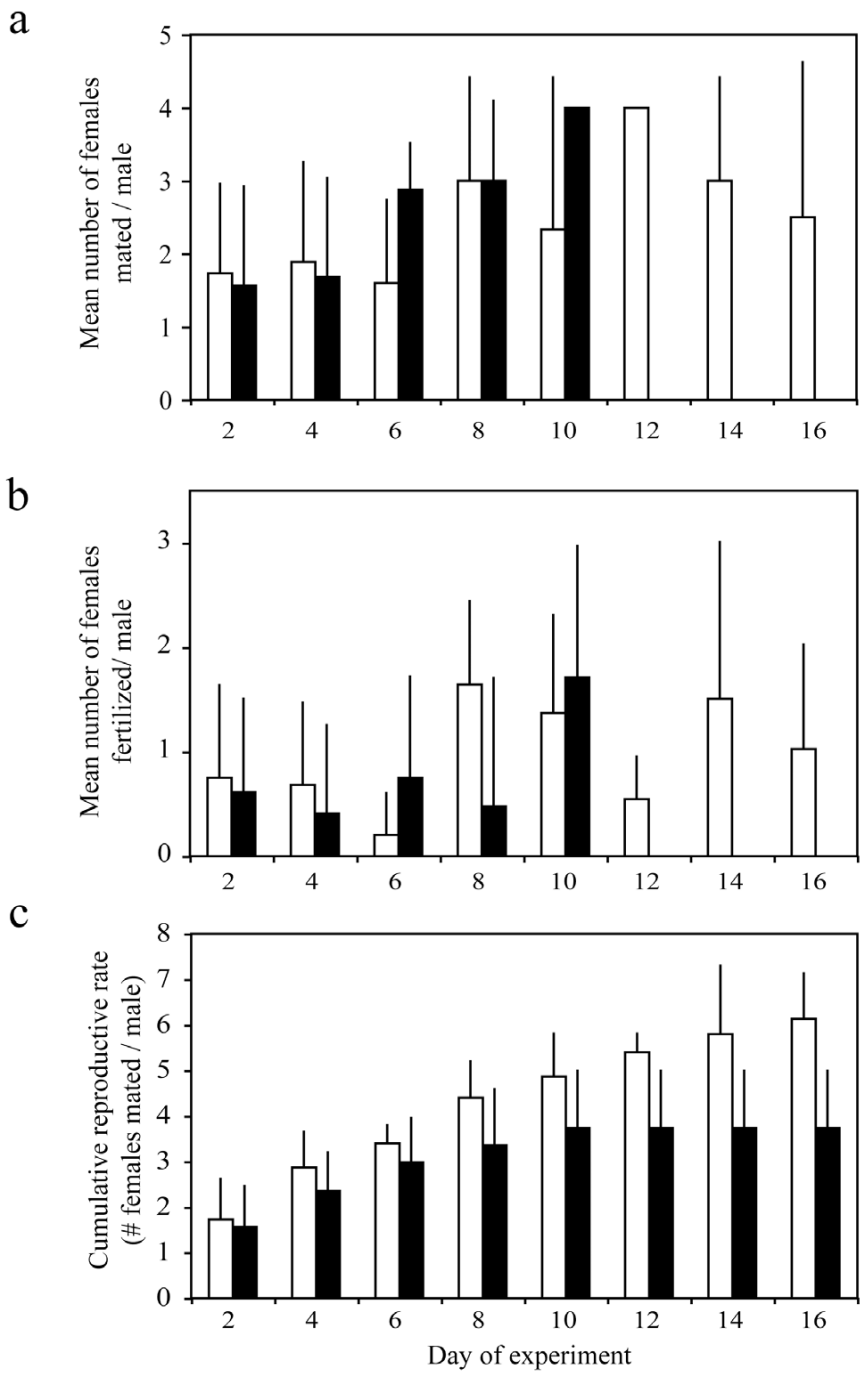
Fecundity of winged and wingless males, a) The average number of females mated by wingless (open bars) and winged (black bars) males on every other day of the experiment, b) The average number of females fertilized by winged and wingless males, c) The relationship between cumulative rate of reproduction (Σl_x_m_x_ where m_x_ = average number of females fertilized/male) and wing morph in individual fecundity experiments. Error bars are +1 SD.

### Mating success of the male wing morphs

Direct observations of male mating behavior were made in arenas that allowed direct competition between a winged and a wingless male for mating with a single virgin female and in cages using several individuals of each morph.

**Figure 2.**
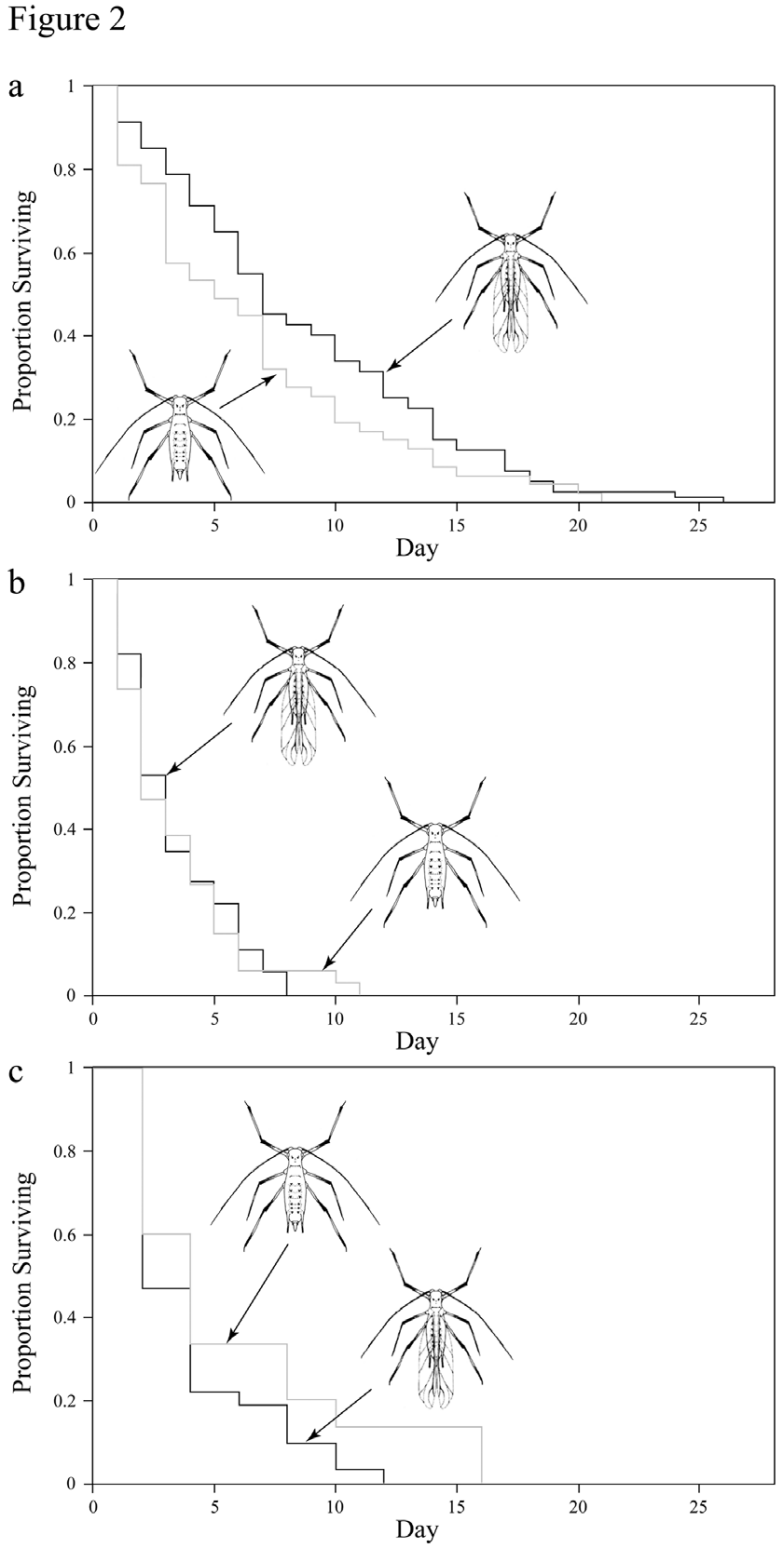
Comparative survivorship of winged and wingless males under different conditions, a) Survivorship of winged (black line) and wingless (grey line) males in food-present conditions, b) Survivorship of winged and wingless males in starvation conditions, c) Survivorship of winged and wingless males in individual fecundity experiments.

### One-on-one competition experiments

Virgin males 24 to 48 hours old of each wing morph were placed with a single virgin female in Medicago plates. Thirty trials were monitored with a time-lapse video camera (one frame/5 seconds) using the QX3+ computer microscope (Intel, www.intel.com/support/intelplay/qx3/index.htm). Mating was monitored for 48 hours. Videos of all trials were analyzed using the software application miXscope version 1.4.1 (Hangstefer 2004). The frequency with which each individual mated and the time until mating was determined. Differences in the frequency of mating between the two male morphs were assessed using Fisher's Exact test.

### Cage experiments

To assess mating success under more natural conditions, a mating experiment was conducted within cylindrical cages measuring 21.5 cm high and 6.5 cm in diameter. In each of six trials, the genitalia of ten winged males and ten wingless males were dusted with a pink or blue powder depending on wing form. The color of the dye was switched between morphs for half of the trials. The marked individuals were placed in a cage containing a small *Medicago sativa* plant with fifteen females and maintained in an incubator (13:11 L:D, 15° C) for four days. At the end of each trial, the number of females mated by each morph was determined by examining the females for dye transfer. The difference in mating success between the two morphs was assessed using a Wilcoxon signed-ranks test (Sokal and Rohlf 1995).

## Results

### Lifetime fecundity experiments

When individual males of each morph were isolated with groups of females, male morphology had no effect on mean daily mating rates (Figure la) (*U* = 100, N_winged_ = 32, N_wingless_ = 14, two-tailed &ntilde;= 0.23) or mean daily fertilization rates (Figure 1b) (*U* = 104, two-tailed p = 0.118). The winged males had a tendency to die earlier than wingless males in this experiment, although this difference was not significant (Figure 1c) (*U* = 284, N_winged_ = 32, N_wingless_ = 15, two-tailed p = 0.31). The cumulative reproductive rate for wingless males was higher than that for winged males, although this result was marginally significant only for fertilization rate (Figure 1c) (*U*, calculation based on mating rate: *U* = 105, p = 0.09; calculation based on fertilization rate: *U* = 108, p = 0.05).

### Comparative survivorship of male wing forms

Winged males survived longer than unwinged males in the presence of food (Figure 2a) (*U* = 2293, N_winged_ = 80, N_wingless_ = *47*, two-tailed p < 0.04). Both winged and wingless males died more quickly in the absence of food than in the presence of food (compare Figures 2a and 2b) (winged males *U* = 3465, N_fed_ = 80, N_starved_ = 55, two-tailed p < 0.001; wingless males *U* = 1095, N_fed_ = 47, N_starved_ = *34*, two-tailed p = 0.004), and there was no significant difference in survival rates between the two morphs when starved (Figure 2b) (*U* = 1004, N_winged_ = 55, N_wingless_ = 34, two-tailed p = 0.56).

Fed winged males (Figure 2a) survived significantly longer than fed winged males that had access to virgin females (Figure 2c) (*U* = 2010, N_winged_ = 80, N_wingless_ = 32, two-tailed p < 0.0001). Fed wingless males (Figure 2a) displayed a trend toward surviving longer than fed wingless males with access to virgin females (Figure 2c), but this difference was not significant (*U* = 404, N_winged_ = 47, N_wingless_ = 15, two-tailed p = 0.4.)

### Mating success of the male wing morphs

#### One-on-one competition experiments

The winged morph was more successful than the wingless morph in procuring matings in an individual rival setting. In 30 mating trials of 48 hrs each, copulations were performed by nine winged males and by one wingless male (Fischer's Exact test; p = 0.006). In two trials the winged male mated multiple times with the same female. Arrival time to the female varied widely. For the winged male, time to copulation ranged from 2 to 1221 min, with a median arrival time of 262 min. Time to copulation for the wingless male was 61 min.

#### Cage experiments

In the four-day cage experiments with several individuals of each male morph, some females were mated by both morphs, some females were mated by only one of the male morphs, and other females were mated by neither morph. There was no difference in mating success between the two morphs (Wilcoxon signed-rank test, p <.5, Table 1). Aphid mortality in these trials averaged over 50% for both winged and wingless males. In the four trials where male mortality data was collected, we found no significant difference in morph survival (Wilcoxon signed-rank test, p <0.75, Table 1).

**Table 1.**
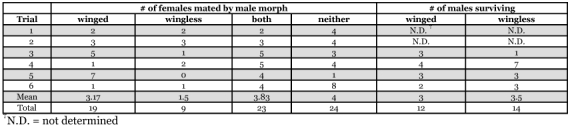
Mating success often winged and ten wingless males in a population cage with fifteen virgin females.

### Discussion

Most studies of the fitness tradeoffs of male wing dimorphism have demonstrated a clear fecundity advantage to winglessness (Crespi 1988; Holtmeier and Zera 1993; Kaitala and Dingle 1993; Langellotto et al. 2000; Novotny 1995; Socha 2004; Socha and Zemek 2004a; Socha and Zemek 2004b; Utida 1972). We found more complicated tradeoffs associated with the wing polymorphism in the male pea aphid.

Winged males obtained 9 out of 10 observed matings when confined in a setting with one-on-one competition between the two morphs. This is in contrast to all other studies, which have reported that flightless males are more aggressive in rival situations (Langellotto et al. 2000; Novotny 1995). It is unclear why winged males were more successful in rival one-on-one competition. Possible explanations include differences in mate sensing and detection, male attractiveness to the female, or time to sexual maturation (Zera and Denno 1997). Variation in time until mating for the winged males was large (1 SD = 501 min), suggesting that mate sensing ability does not play a role in mating success. But because we observed only one mating of a wingless male, we could not accurately compare average time to arrival for each morph. The individual fecundity experiments show that the earliest mating and fertilization rates for the two wing morphs are not significantly different (Figure 1a and 1b), so it is unlikely that adult winged males reach sexual maturity before adult wingless males. In addition, although pre-copulatory female guarding has been described in several other aphid species (Foster and Benton 1992), no overt male guarding was observed, such as perching on the dorsum of the female for extended periods of time. The possibility that the winged males performed more subtle forms of guarding cannot be excluded.

The simplest explanation for the winged male's advantage in rival mating situations is that this morph is more active than the wingless male. While the winged male moved often, it was not unusual for the wingless male to remain stationary during the entire trial of 48 hours. The heightened activity of the winged males may be a side effect of a migratory tendency.

The clear mating advantage of the winged males was lost in the second assay of reproductive fitness in the cage experiments. Although there seemed to be a slight tendency for the winged males to mate more frequently than their wingless counterparts in these competition experiments (Table 1), this difference was not statistically significant. Examining the differences between the rival one-on-one experiments and the cage experiments may help elucidate the mechanisms that underlie differential mating success based on wing form. For example, the effect of mate sensing ability may be reduced in the cage experiment due to the longer experimental duration and more complex plant architecture. In longer trials, time to mating may be irrelevant, especially if males do not mate preferentially with virgins. Differential survival of the two morphs may have also affected mating success in the cage experiments. Average male mortality during these experiments was over 50%, suggesting that male survivorship could have had an effect on the number of females mated. Although the final assessment of male mortality from this experiment was not significantly correlated with wing morph (Table 1), one morph may have consistently died earlier during the four day experimental period. This would skew the final evaluation of mating success.

This last possibility seems likely since the results from the other experiments indicate that survivorship is associated with wing form. When food was present, winged males survived significantly longer than wingless males (Figure 2a). Under starvation conditions, both morphs died at approximately the same rate (Fig 2b). Reproductive activity dramatically reduced the survivorship of both male morphs. The mean lifespan of males in individual fecundity experiments (Figure 2c) was almost as short as male lifespan assessed in starvation conditions. There was a tendency for the wingless aphids to outlive the winged aphids during reproductive activity. Because sample size for the individual fecundity experiments was small, especially near the end of the survival curve, this trend was not significant.

The tendency for the winged males to die sooner when sexually active had a significant effect on the net reproductive rate. In the absence of competition, wingless males had significantly higher net reproductive output than winged males (Figure 1c). Both winged and wingless males showed no evidence for a decline in fertilization success with time (Figures 1a, b). There is therefore no evidence from these experiments that males are sperm limited.

It is not clear whether the subtle differences we observed in the mating ability of winged and wingless males were associated with the intrinsic costs of flight apparatus maintenance. Some studies of wing polymorphism in male insects provide evidence that flight capacity in males, including production and maintenance of flight musculature (Socha and Sula 2006), has the same effect on reproductive success as it does in females. However, the slight differences in reproductive fitness that was measured in winged and wingless pea aphids did not appear to be directly related to the physiological distribution of resources. For example, when both winged and wingless males were dissected, no obvious differences in the size or structure of the testes were observed (unpublished data). Our experiments addressed only events that occurred when males and females were already in the same location. Under these circumstances neither morph appeared to have a clear advantage. However, winged males presumably encounter increased risk of not finding a mate when they fly. There must, therefore, be a countervailing advantage for the winged form. It is possible that the advantages of producing winged morphs accrue from the benefits of outbreeding.
